# MetAAA Trial Patients Receiving Metformin Therapy Show Limited Improvement in Quality of Life Compared to AAA Patients with Placebo Intake—A Double-Blind, Randomized, and Placebo-Controlled Trial

**DOI:** 10.3390/medsci13040273

**Published:** 2025-11-15

**Authors:** Johannes Klopf, Robin Willixhofer, Diana Ahmadi-Fazel, Andreas Scheuba, Lukas Fuchs, Anna Sotir, Anders Wanhainen, Christine Brostjan, Christoph Neumayer, Wolf Eilenberg

**Affiliations:** 1 Department of General Surgery, Division of Vascular Surgery, University Hospital Vienna, Medical University of Vienna, 1090 Vienna, Austria; johannes.klopf@meduniwien.ac.at (J.K.); robin.willixhofer@medunwien.ac.at (R.W.); diana.ahmadi-fazel@meduniwien.ac.at (D.A.-F.); andreas.scheuba@meduniwien.ac.at (A.S.); lukas.fuchs@meduniwien.ac.at (L.F.); anna.sotir@meduniwien.ac.at (A.S.); christine.brostjan@meduniwien.ac.at (C.B.); christoph.neumayer@meduniwien.ac.at (C.N.); 2 Department of Surgical Sciences, Uppsala University, 75185 Uppsala, Sweden; anders.wanhainen@uu.se; 3 Department of Surgical and Perioperative Sciences, Umeå University, 90185 Umeå, Sweden

**Keywords:** health-related quality of life (QoL), abdominal aortic aneurysm (AAA), patient-reported outcome measure, metformin

## Abstract

**Background:** Abdominal aortic aneurysm (AAA) is a multifactorial vascular disease with limited therapeutic options, as no pharmacological treatments currently exist to mitigate the progression of small AAAs. Quality of life (QoL) has emerged as a valuable supplementary metric for assessing the efficacy of pharmacological interventions. This study evaluated QoL scores of MetAAA trial patients on metformin therapy compared to those with placebo intake. **Methods:** Overall, 54 patients with AAA were included in the MetAAA trial (ClinicalTrials.gov-Identifier:NCT03507413) and randomized to either metformin or placebo treatment. All participants were asked to complete three established and validated (in total 659 longitudinally collected) QoL questionnaires: (1) the 36-Item Short Form Health Survey (SF-36), (2) the Aneurysm Symptom Rating Questionnaire (ASRQ), and (3) the Aneurysm-Dependent Quality of Life questionnaire (ADQoL). **Results:** A superior health-related QoL was found in metformin-treated AAA patients compared to enrolled AAA patients receiving a placebo. In detail, AAA patients undergoing metformin treatment showed a superior overall current QoL score (*p* = 0.038), general health perception (*p* = 0.013), improved physical functioning (*p* = 0.004), and increased energy/lower fatigue scores (*p* = 0.008). Furthermore, fewer limitations due to cognitive distress (*p* = 0.001) and lower limb function (*p* = 0.021) were detected. Other QoL subscales did not show statistical significance. Inflammatory blood parameters suggest that while systemic inflammation may have some impact on perceived QoL, the relationship is largely limited. **Conclusions:** In patients with small AAA, metformin led to a limited improvement in health-related QoL compared to a placebo.

## 1. Introduction

An abdominal aortic aneurysm (AAA) is a common condition that can result in increased morbidity or, in more severe cases, if ruptured, considerable mortality [1,2,3]. AAA are commonly diagnosed incidentally, are a leading cause of mortality in developed countries, and their associated morbidity, mortality, and cost are significant public healthcare burdens [4]. Modifications in hemodynamic factors, atherosclerosis, and persistent aortic inflammation are recognized as contributors to the development and progression of an AAA [5,6,7]. Since AAA progress over time, current clinical guidelines recommend regular imaging surveillance. To date, the maximal aortic diameter remains the sole parameter used clinically to predict aneurysm growth, rupture risk, and the need for surgical intervention [2,8,9]. Particularly given the current absence of a specific pharmacological therapy for AAA, surveillance, treatment for cardiovascular risk reduction and new therapeutic approaches tested in clinical studies may influence patients’ health-related quality of life (QoL) [10]. Metformin is being investigated as a promising potential treatment option for AAA in clinical studies. Conducted as a prospective, double-blind, randomized, placebo-controlled study, the Vienna MetAAA trial investigated the safety and therapeutic efficacy of oral metformin therapy in non-diabetic patients with AAA. No differences in AAA progression were observed between the metformin and placebo groups [11]. Additionally, the Swedish Metformin for Abdominal Aortic Aneurysm Growth Inhibition (MAAAGI) trial, an open-label randomized controlled trial, seeks to provide further supporting evidence for this therapeutic approach [12]. However, we have previously shown that participants in the MetAAA trial reported superior QoL compared to patients undergoing standard surveillance for small AAA [13]. Hence, metformin and other emerging therapies may positively impact QoL in individuals with AAA. Beyond traditional endpoints like morbidity and mortality, subjective measures, including health-related QoL and patient satisfaction, provide valuable insights into patients’ well-being and overall health condition [14]. Notably, patients with AAA demonstrate a high willingness to participate in clinical trials, and recent studies found no evidence that AAA surveillance negatively affects QoL [15]. Nevertheless, factors such as potential treatment delays, limited coping capacity, required lifestyle adjustments, and sustained emotional stress, whether chronic or related to preoperative anxiety, can promote lasting behavioral changes in AAA patients and negatively impact their QoL [16,17]. Quality of life has grown in consideration, not only used to evaluate the outcomes of surgical AAA treatment, but also as a helpful, supplementary metric in studies testing the efficacy of pharmacological interventions [18]. In pharmacological AAA studies, QoL is a clinically relevant endpoint, complementing traditional measures such as aneurysm growth or rupture risk. Even a small AAA can impair physical and emotional well-being, and including QoL endpoints allows for assessment of how interventions affect patient-centered outcomes beyond anatomical measures [19]. How participation in double-blind, randomized, and placebo-controlled clinical trials (such as MetAAA), and the possible effect of the study intervention’s treatment with either metformin or placebo, affect QoL is unclear and is the topic of this study. Metformin may improve QoL via both biological and psychosocial pathways. Beyond its pleiotropic metabolic effects, metformin has been shown to reduce vascular and systemic inflammation [20,21,22,23]. These anti-inflammatory and vasculoprotective actions could enhance physical energy and reduce fatigue, while improvements in metabolic homeostasis may support better mood and cognitive functioning [24,25,26]. Thus, these combined physiological and psychosocial mechanisms provide a plausible basis for improved patient-reported QoL in chronic disease settings.

Employing established and validated instruments, we proposed that participants receiving metformin in the MetAAA trial would demonstrate superior QoL outcomes than those in the placebo cohort.

## 2. Methods

### 2.1. MetAAA Trial

**Figure 1:**
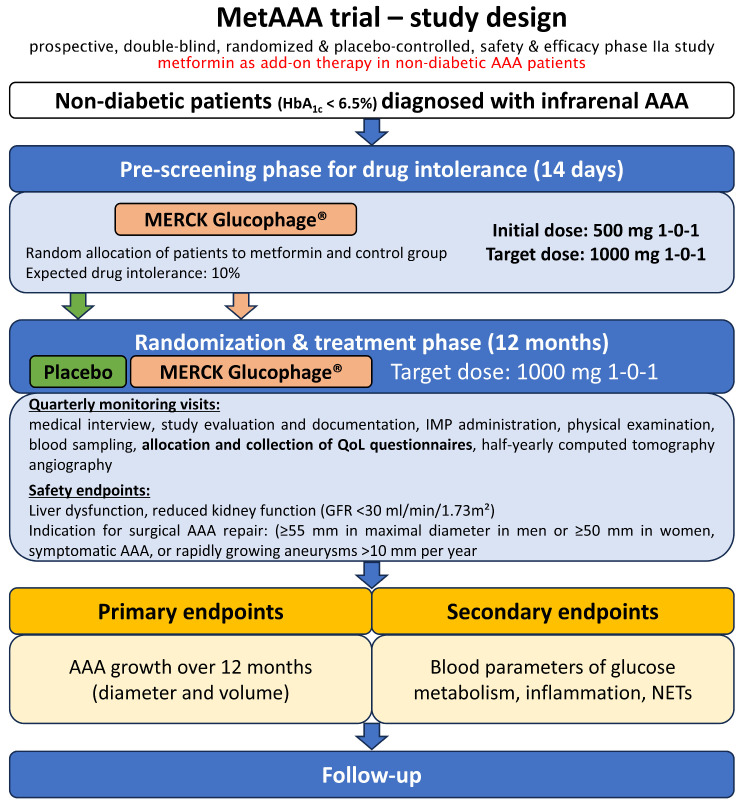
**Overview of the MetAAA trial study design**. Adapted from Klopf et al. [13]. In the MetAAA trial, metformin was investigated in a prospective, randomized, double-blind, placebo-controlled study to evaluate its safety and therapeutic efficacy. Patients identified with an infrarenal AAA measuring 30–49 mm on computed tomography angiography were enrolled following the screening phase and subsequently assigned to treatment with either metformin or placebo. Quarterly monitoring visits during the twelve months of treatment and subsequent half-yearly follow-up visits were additionally used to allocate and collect established and validated questionnaires for the assessment of QoL. Abbreviations: AAA, abdominal aortic aneurysm; IMP, investigational medicinal product, QoL, quality of life; GFR, glomerular filtration rate; NETs, neutrophil extracellular traps.

The MetAAA trial, initiated at the Medical University of Vienna, started in September 2018 and has currently prematurely ended follow-up due to slow recruitment during the COVID-19 pandemic. This study is registered in the clinical trials registry (ClinicalTrials.gov Identifier: NCT03507413) and was conducted as a prospective, randomized, double-blind, placebo-controlled phase IIa study. This trial investigated the safety and efficacy of oral metformin therapy versus a placebo in non-diabetic individuals with AAA. Supported by preclinical and observational evidence from diabetic AAA cohorts, the MetAAA trial evaluated the therapeutic efficacy of oral metformin therapy for disease control in non-diabetic patients with AAA [27,28,29,30,31]. Thus, the MetAAA trial was primarily designed to identify the first medical therapy that could inhibit AAA progression, with the goal of minimizing the need for surgery and reducing the risks and costs related to aneurysm rupture, including mortality and morbidity. We have previously shown that participants in the MetAAA trial report superior QoL compared to patients undergoing standard surveillance for small AAA [13]. Figure 1 depicts an overview of the MetAAA trial study design. All participants in the MetAAA trial were treated with metformin or placebo for twelve months, with a daily dose of 2000 mg (two 500 mg tablets twice daily), related to the established treatment efficacy in diabetes mellitus. Serum creatinine was serially assessed at baseline and during follow-up to monitor renal function and ensure metformin’s safety. Ethical conduct for this human-subject study adhered to the 2013 revision of the Declaration of Helsinki and was in line with the CONSORT-Outcomes 2022 extension and approved by the institutional ethics committee of the Medical University of Vienna (license no. 1479/2017, approval date: 30 November 2017) [32].

### 2.2. Study Population

**Figure 2:**
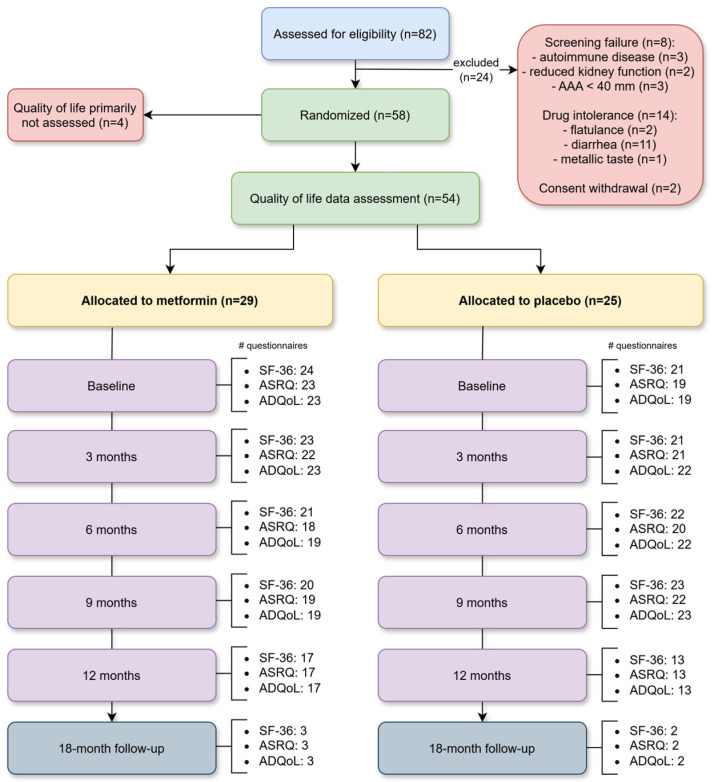
**Flow chart**. Flow diagram illustrating participant enrollment, randomization, allocation, and follow-up as well as quality of life questionnaire distribution in the MetAAA trial. Abbreviations: AAA, abdominal aortic aneurysm; SF-36, 36-Item Short Form Health Survey; ASRQ, Aneurysm Symptom Rating Questionnaire; ADQoL, Aneurysm-Dependent Quality of Life questionnaire.

Fifty-four non-diabetic MetAAA trial participants (10 women and 44 men) with an infrarenal AAA with a maximal aortic diameter of between 30 and 49 mm were included (Figure 2). All MetAAA trial participants gave their written informed consent and were treated and followed-up at the outpatient clinic of a tertiary university hospital (University Hospital Vienna). Independent enrollment of study participants was performed, and the trial’s exclusion criteria were diabetes mellitus (percentage glycosylated hemoglobin (HbA_1c_) ≥ 6.5% (48 mmol/mol blood glucose), indication for surgical AAA repair (≥55 mm in maximal diameter in men or ≥50 mm in women, for symptomatic or rapidly growing aneurysms (greater than 10 mm per year), pregnancy and contraindications for metformin (most importantly severe renal impairment (eGFR < 30 mL/min/1.73 m^2^), acute or chronic liver dysfunction with metabolic acidosis, and hypersensitivity to metformin) [2,8,9,33,34,35,36]. Patients with prediabetes—HbA_1c_ range of ≥5.7–6.4% (39–46 mmol/mol blood glucose were included in this study. Demographic characteristics were obtained through a structured questionnaire, and all study participants completed serial follow-up visits that incorporated validated QoL assessments.

### 2.3. Questionnaires and Quality Assessment

All participants underwent assessment through three validated questionnaires during their scheduled monitoring visits (at baseline, then quarterly throughout the 12-month treatment period, and once following the half-yearly follow-up visit). At first visit (before randomization), all MetAAA trial participants received one-on-one guidance, assistance, and support provided by the same attending study physician (resident at the Division of Vascular Surgery, specialized in vascular surgery and supervised by a consultant professor for vascular surgery). Furthermore, a study nurse assisted participants in accurately and fully completing the validated questionnaires, if necessary. Following that, the participants were advised to complete the comprehensive questionnaires at home to ensure that they were answered in a quiet and patient-centered setting and subsequently submit them to the study center. Once received, the questionnaires were reviewed in an interview to clarify any unresolved issues. The following validated questionnaires were included: the 36-Item Short Form Health Survey (SF-36), the Aneurysm Symptom Rating Questionnaire (ASRQ), and the Aneurysm-Dependent Quality of Life questionnaire (ADQoL) [37,38,39,40,41]. Each questionnaire depended on patient self-reporting and independent completion. Comprising eight health concepts, the SF-36 is a widely used set of generic QoL measures that can be readily administered and capture the overall health status of the patient. The eight health concepts are: general health perception, physical functioning, bodily pain, role limitations due to physical health problems, role limitations due to personal or emotional problems, emotional well-being, social functioning, and energy/fatigue. Designed to assess symptoms associated with AAA, the ASRQ measures their effects on patients and records treatment experiences throughout the surveillance period. The ASRQ includes 44 items across six subscales: general malaise, weight, emotional, lower limb, cognitive, and gastrointestinal symptoms. The ADQoL, a 24-item questionnaire, measures individualized QoL and the disease-specific impact of AAA on patients’ QoL. Since the ADQoL is designed to be an individualized measure, the patients can weigh the ratings of the impact of AAA on each aspect of their lives to reflect a highly personalized assessment of the QoL impact of AAA. For the ADQoL, four subscales are implemented: physical function, psychological health, social life, and a subscale with non-assignable items.

### 2.4. Analysis of Inflammatory Blood Parameters

Inflammatory blood parameters such as leukocytes, C-reactive protein, and D-dimer were obtained at every patient visit as part of a routine set of diagnostic tests available from the Vienna University Hospital central laboratory service. Additionally, plasma myeloperoxidase (MPO) levels and a calculated combined MPO/D-dimer score were measured using ELISA as per manufacturer’s instructions (R&D Systems, Minneapolis, MN, USA) using a previously described protocol and publication [42].

### 2.5. Statistical Methods

Every set of patient-reported outcome measure (PROM) questionnaires were analyzed in accordance with their formal and recommended evaluation methodology. To analyze the SF-36 sets, graded answers were transformed and normalized depending on presence or absence of limitations (Supplemental Table S1) and mean values were calculated for the eight health concepts. For the ASRQ and ADQoL sets, recorded values were converted (Supplemental Table S1) and PROMs, if applicable, were pooled (Supplemental Table S2). Continuous variables of demographic data are reported as median and interquartile range (IQR), while categorical variables are expressed as counts and percentages. Validated questionnaire results are expressed as mean values and standard deviation (SD) for continuous variables and as absolute and relative frequencies for categorical data. Additionally, ADQoL outcomes are presented as dimensionless weighted factors. For assessment of statistical significance, non-parametric tests were employed (Mann-Whitney U test and χ^2^ test). To compare repeatedly measured QoL scores between the study groups, the repeated measurements were first averaged within each subject. The resulting average scores were described by median and interquartile range and compared between groups using Wilcoxon rank sum tests. Due to the exploratory nature of the study (with QoL as predefined exploratory endpoint), no adjustment for multiple testing was applied and *p* values are presented descriptively. Global analysis was employed to address possible cohort differences, such as incomplete baseline or follow-up data, while avoiding the necessity of sensitivity analyses or multiple measurement correction. Missing or partially completed questionnaires were analyzed according to the evaluation guidelines of each instrument. Missing items were omitted from subscale and total score calculations without data imputation. Analysis showed no systematic differences between groups; therefore, missingness was considered random. The study was powered to detect differences in the MetAAA trial regarding the primary endpoint of AAA growth rates; however, it was not specifically powered to detect differences in QoL or any biomarker outcomes. No separate sample size calculation was conducted, which may have reduced the statistical power to detect between-group differences.

## 3. Results

**Patients.** In total, fifty-four individuals participating in the MetAAA trial were enrolled in this study. Among them 25 participants received placebo and 29 were under metformin intake. Detailed demographic information for both groups can be found in Supplemental Table S3. Due to recruitment challenges during the COVID-19 pandemic, enrollment of the main trial was stopped after 58 randomized patients and the study proceeded to data analysis. Baseline data were available for all participants (Supplemental Table S4, intention to treat analysis). Since QoL was later added as a secondary outcome, four patients did not have QoL data assessment. Figure 2 illustrates the flow diagram with participant enrollment, randomization, allocation and follow-up as well as quality of life questionnaire distribution in the MetAAA trial.

**Compilation of QoL questionnaires.** Analysis included 659 QoL questionnaires, of which 507 (76.93%) were complete and 152 (23.07%) were incomplete, leading to an overall response rate of 81.06%. Among all questionnaires, 327 (49.62%) and 332 (50.38%) were from study participants receiving placebo or metformin, respectively. Detailed information on the distribution and collection of QoL questionnaires at each monitoring timepoint is provided in Supplemental Table S5.

**MetAAA trial patients receiving metformin show increased health-related QoL.** The analysis of the SF-36 health concepts revealed superior health-related QoL dimensions of MetAAA trial patients receiving metformin compared to participants with placebo intake, as depicted in Table 1. Among the eight SF-36 health concepts, AAA patients on metformin therapy rated their general health significantly higher than placebo-treated participants (61.53 vs. 56.49%, *p* = 0.019). Moreover, energy/fatigue (62.63 vs. 54.72%, *p* = 0.037) was detected to show a significant difference indicating superior QoL in MetAAA trial patients receiving metformin.

The analysis of the ASRQ (Table 2) revealed no significant differences regarding the appearance of negative symptoms between placebo- and metformin-treated MetAAA trial patients. Evaluation of symptom-induced limitations using the ASRQ indicated that metformin-treated AAA patients exhibited significantly fewer lower limb restrictions than those receiving the placebo (1.75 vs. 0.12%, *p* = 0.021). Also, fewer limitations due to cognitive distress were detected in metformin-treated study participants (1.00 vs. 0.46%, *p* = 0.042).

All ASRQ response frequencies of patient-reported outcome measures for participants in the MetAAA trial, separated by the placebo and metformin treatment groups, are detailed in Supplemental Table S6. The ADQoL, serving as an individualized measure of current QoL and associated aneurysm-specific impact displayed a significant difference for study participants under metformin treatment for higher current QoL (3.49 vs. 3.83, *p* = 0.038) compared to patients under placebo intake (Table 3). The individual evaluation of ADQoL patient-reported outcome measures is presented in Supplemental Table S7. Comparisons of SF-36 health concepts, ASRQ, and ADQoL subscales between MetAAA trial participants receiving placebo or metformin are shown at baseline in Supplemental Table S8 and after twelve months in Supplemental Table S9.

**Blood parameters.** The inflammatory blood biomarkers did not show significant differences between the metformin and placebo groups at baseline or after twelve months of treatment. Leukocytes (R = 0.270, *p* = 0.049) and C-reactive protein (R = 0.314, *p* = 0.021) demonstrated weak correlations with overall current QoL score derived from ADQoL subscales (lower score values for weighted indices indicate superior quality of life) (Supplemental Table S10). Moreover, plasma levels of myeloperoxidase showed inverse correlations with ADQoL subscales of physical function (R = −0.409, *p* = 0.002) and social life (R = −0.284, *p* = 0.039) (higher score values for weighted indices indicate superior quality of life). No further correlations were found between the inflammatory biomarkers and the ADQoL subscales, SF-36 health concepts, or ASRQ subscales.

## 4. Discussion

In this single-center randomized trial, 659 validated QoL questionnaires from 54 AAA patients (29 on metformin, 25 on placebo) were analyzed, revealing that metformin-treated participants showed an overall superior current QoL score and exhibited superior overall health perception and higher energy levels with reduced fatigue. Additionally, these patients experienced fewer limitations related to cognitive distress and lower limb function.

A systematic review integrating both quantitative and qualitative studies found that AAA surveillance does not adversely affect QoL [15]. In a recent study, we additionally demonstrated that MetAAA trial patients show superior QoL compared to patients under regular surveillance for small AAA, indicating the importance of clinical trial participation for AAA patients [13]. Participation in AAA surveillance may already enhance QoL when patients receive new interventions, like metformin for AAA growth management, potentially amplified by the Hawthorne effect associated with increased clinical oversight. While the diagnosis of an AAA can negatively affect QoL, it has been shown that AAA patients generally feel secure under surveillance and major apprehensions result from insufficient disease information [15,19]. The United Kingdom Aneurysm Growth Study reported a transient reduction in mental (not physical) QoL scores following AAA diagnosis compared to non-aneurysmal study participants, with physical QoL scores remaining consistently lower, even after twelve months [43]. Notably, our research revealed that AAA patients receiving metformin treatment encountered significantly fewer limitations due to lower limb dysfunction and overall displayed an improved physical QoL score. Except for bodily pain, fluctuations in mental and physical QoL aspects were found to be dependent on exercise dosage, pointing to a higher likelihood of QoL improvement through enhanced energy and alleviated symptom limitations [44]. Following that, as our study also revealed increased energy levels, less fatigue, and fewer limitations due to cognitive distress in metformin-treated AAA patients, it may seem that QoL improvement in this study is more reliant on physical health than mental health parameters. This is also supported by a cohort study, which demonstrated that a combined therapy of exercise and metformin was most effective in long-term therapies to improve QoL in patients with diabetes mellitus type II [45]. QoL improvements were mainly observed in physical health domains, reflecting patient-reported perceptions of energy, fatigue, and mobility, which may not directly correspond to objective functional measures. Such discrepancies are common in older adults and AAA patients, underscoring the importance of patient-reported outcomes [15,46]. Future studies should include objective functional assessments, such as walking distance or exercise tolerance, to corroborate QoL improvements and clarify their physiological relevance. Although various QoL scales assessed in this study are common in older populations, metformin may offer broader benefits beyond aneurysm-specific effects. Interestingly, a multicenter study showed that after adjustment for age, sex, body mass index, physical activity, and antihypertensive and antihyperlipidemic treatment, patients with diabetes mellitus type II are associated with reduced SF-36 health concept scores (except for mental health) and consequently reduced quality of life [47]. However, this study did not observe the improving effects on QoL of metformin compared to other antidiabetic medication. A recent study revealed that in individuals with advanced heart failure with reduced ejection fraction and diabetes mellitus type II, the administration of metformin was linked to favorable outcomes and improved QoL, extending beyond its role in blood glucose regulation [48]. Other studies describe a beneficial effect on patient-reported QoL in patients with gestational diabetes and diabetes mellitus type I, both under metformin prescription [49,50]. Since AAA is considered an inflammatory disease, it is notable that metformin significantly improved inflammation, disease severity, and QoL in patients with rheumatoid arthritis in a randomized controlled study [51]. Metformin, a pleiotropic drug targeting the proinflammatory pathways involved in AAA progression, has also shown efficacy in reducing clinical flares, prednisone use, and body weight in a proof-of-concept trial for mild to moderate systemic lupus erythematosus [52,53]. Metformin’s benefits in other disease processes, such as diabetes mellitus and cardiovascular health, have been well-documented, and this study tried to detect potential broader applications for improving QoL in non-diabetic AAA patients. The observed improvement in QoL, particularly within physical health domains, may be mediated by metformin’s multifaceted biological and psychological effects [20,23]. Metformin enhances metabolic efficiency and mitochondrial function while reducing vascular inflammation through AMPK activation and NF-κB inhibition [21,54]. Furthermore, metformin stabilizes glucose metabolism, which may enhance energy balance, improve physical vitality and cognitive performance, and reduce fatigue perception [55]. Beyond these physiological mechanisms, treatment with metformin may foster a sense of proactive disease management and vitality, thereby contributing to better emotional well-being and patient-reported QoL. Together, these interlinked pathways may provide a plausible explanation basis for the improvements in QoL seen in our trial’s metformin group.

Although this study indicates limited improved QoL in MetAAA patients, similar QoL improvements are commonly seen across randomized trials, particularly in oncology, where they are valued as positive outcomes and may even hold prognostic relevance due to trial participation [56,57]. The results of this study suggest a subtle link between inflammatory blood parameters and QoL in AAA patients, though low or normal baseline biomarker levels may have limited the detection of significant effects over time. Despite no detected significant differences in inflammatory biomarkers between the metformin and placebo groups during the study course, weak correlations were observed between leukocytes, C-reactive protein, and the overall current QoL. These findings imply that while systemic inflammation may have some effect on the perceived quality of life, the relationship appears to be very limited. Furthermore, higher plasma levels of myeloperoxidase, a marker of neutrophil activation, might be associated with poorer QoL in specific domains, particularly those related to physical health and social interaction. However, the absence of significant correlations with other QoL measures, such as the SF-36 health concepts or ASRQ subscales, indicates that inflammation may not uniformly impact all aspects of patient-reported outcomes.

Study Limitations: Since QoL data was retrieved as an additional measure of the MetAAA trial, this study’s sample size and attrition rate certainly limited the ability to detect further QoL or biomarker differences between AAA patient groups, reducing the generalizability of the findings. The study was powered to assess differences in the primary endpoint of AAA growth rates in the MetAAA trial; no separate sample size calculation was performed for QoL or biomarker outcomes, which may have reduced its statistical power to specifically evaluate differences in QoL or biomarker outcomes. Although some QoL differences between groups reached statistical significance, the absolute effect sizes appear limited. The lack of established minimally clinically important difference thresholds for the PROMs used in AAA patients made it challenging to determine the clinical relevance of the potentially observed differences. Nevertheless, in patients with AAA, typically older and burdened by comorbidities, even modest improvements in perceived physical and mental health may translate into meaningful benefits for daily functioning and well-being. While 12-month treatment with metformin was assessed, the follow-up period of 6 more months may not be long enough to capture the long-term effects or delayed outcomes of the treatment. Current clinical practice guidelines suggest metformin as a prospective candidate medication for AAA treatment, particularly in the context of clinical trials [2,9]. Randomized controlled trials are essential for clinical practice, but evidence on conservative aneurysm treatment and its effect on the QoL of AAA patients remains very limited [58]. Three validated QoL questionnaires, two of which were specifically developed to assess and evaluate QoL in patients with AAA, were utilized in this study. Patients provided the data via self-report and independent questionnaire completion [37,38,39,40,41]. A total of 659 QoL questionnaires were analyzed, with 507 (76.93%) fully completed and 152 (23.07%) partially completed. A recent systematic review of 811 studies found an average response rate in patient surveys in surgery of 70%, while this study achieved a significant higher rate of 81.06%, likely due to direct engagement, such as face-to-face interactions, clinical study design, and one-on-one support during monitoring visits [59]. Despite comprehensive information and support, 23.07% of questionnaires were incomplete, with most missing only 1–5.40% of questions. Among the partial responses, 74.25% of unanswered questions related to work or sex life, which were particularly challenging for the older AAA patient group, and questions about finances and future outlook were often left blank. The observed QoL scales may partly reflect a Hawthorne effect, as study participation itself can influence patient-reported outcomes. However, the double-blind, placebo-controlled design minimizes potential placebo or expectancy bias, suggesting that such non-specific effects are unlikely to fully explain the findings. While underreporting bias and missing data may reduce statistical power, this was mitigated by ensuring confidentiality, providing support, and using validated questionnaires with comprehensive statistical analysis [60].

Future studies should pursue multicenter validation to determine whether QoL benefits under metformin treatment generalize beyond our single-center cohort. Extended follow-up is needed to assess long-term persistence of improvements and potential delayed effects on AAA progression. Mechanistic studies are warranted, given that QoL improvement occurred without apparent changes in systemic inflammatory biomarkers.

## 5. Conclusions

Based on the results of this study with patients with small AAA, metformin therapy appears to be associated with a slight improvement in health-related QoL; however, this finding requires confirmation in larger, multi-center, and long-term studies.

## Figures and Tables

**Table 1:** SF-36 health concept comparison between metformin- and placebo-treated AAA patients in the MetAAA trial, reflecting health-related QoL.

	MetAAA Trial Cohort Placebo		MetAAA Trial Cohort Metformin		*p* Value
***SF-36—Health concepts***	*n*	Mean [Score] (SD)	*n*	Mean [Score] (SD)	
*General health perception*	**25**	**56.49 (16.68)**	**29**	**61.53 (18.79)**	**0.019**
*Physical functioning*	25	63.01 (20.81)	29	68.29 (29.27)	0.302
*Bodily pain*	25	73.90 (26.22)	29	75.41 (28.80)	0.529
*Role limitations due to physical health problems*	25	53.24 (41.17)	29	41.17 (45.20)	0.599
*Role limitations due to personal or emotional problems*	25	71.60 (40.20)	29	72.57 (40.39)	0.533
*Emotional well-being*	25	73.10 (20.54)	29	76.22 (21.22)	0.151
*Social functioning*	25	79.59 (22.16)	29	82.83 (23.56)	0.226
*Energy/fatigue*	**25**	**54.72 (19.27)**	**29**	**62.63 (21.84)**	**0.037**
*Health change over recent 12 months*	25	46.43 (17.30)	29	49.31 (15.22)	0.149

Abbreviations: AAA, abdominal aortic aneurysm; SF-36, 36-Item Short Form Health Survey; n, number of patients; SD, standard deviation. Scores represent normative values of the SF-36 health concepts. The data were analyzed with the Mann-Whitney U and Wilcoxon rank-sum tests. Bolded results denote statistically significant differences.

**Table 2:** ASRQ subscale comparison between metformin- and placebo-treated AAA patients in the MetAAA trial, reflecting health-related QoL.

	*MetAAA Trial Cohort* *Placebo (25 Patients)*		*MetAAA Trial Cohort* *Metformin (29 Patients)*		*p Value*
***ASRQ subscales***					
***Appearance of negative symptoms***	*n*/*N* of items	%	*n*/*N* of items	%	
*General malaise*	378/956	39.54	370/972	38.07	0.507
*Weight*	110/319	34.48	121/324	37.35	0.449
*Emotional*	349/942	37.05	339/946	35.84	0.372
*Lower limb*	384/956	40.17	377/972	38.79	0.535
*Cognitive*	252/639	39.44	241/646	37.31	0.432
*Gastrointestinal*	307/850	36.12	335/862	38.86	0.233
*Total appearance of negative symptoms*	1725/4494	38.38	1689/4506	37.48	0.197
*Other negative symptoms (non-assignable)*	7/30	23.33	9/35	25.71	0.824
***Limitations due to symptoms***	*n* (of questionnaires)	Median [%] (IQR)	*n* (of questionnaires)	Median [%] (IQR)	
*General malaise*	106	1.50 (2.00)	109	1.68 (1.92)	0.549
*Weight*	106	0.25 (0.80)	109	0.00 (0.70)	0.647
*Emotional*	106	2.00 (2.25)	109	1.00 (2.08)	0.519
*Lower limb*	**106**	**1.75 (3.00)**	**109**	**0.12 (1.03)**	**0.021**
*Cognitive*	**106**	**1.00 (2.25)**	**109**	**0.46 (0.93)**	**0.042**
*Gastrointestinal*	106	1.00 (1.17)	109	0.83 (1.57)	0.394
*Total limitations due to symptoms*	106	10.00 (7.50)	109	7.38 (5.62)	0.128

Abbreviations: AAA, abdominal aortic aneurysm; ASRQ, Aneurysm Symptom Rating Questionnaire; n, number of applicable items; N, total number of items; SD, standard deviation. Scores represent the percentage of total possible score achieved. The data were analyzed with the χ^2^ and Wilcoxon rank sum tests. Bolded results denote statistically significant differences.

**Table 3:** ADQoL subscale comparison between metformin- and placebo-treated AAA patients in the MetAAA trial, reflecting health-related QoL.

	*MetAAA Trial Cohort* *Placebo*		***MetAAA Trial Cohort*** *Metformin*		*p Value*
***ADQoL subscales***	*n*	*WI (SD)*	*n*	*WI (SD)*	
*Physical function*	25	−1.58 (1.99)	29	−1.20 (2.31)	0.708
*Psychological health*	25	−1.04 (2.25)	29	−0.75 (2.14)	0.357
*Social life*	25	−0.45 (1.66)	29	−0.58 (1.85)	0.924
***Individual evaluation***	***MetAAA trial cohort*** ***Placebo***		***MetAAA trial cohort*** ***Metformin***		***p Value***
	*N*	*Median (IQR)*	*N*	*Median (IQR)*	
*Current QoL (I)*	**25**	**3.83 (0.75)**	**29**	**3.49 0.91)**	**0.038**
*QoL if I would not have an AAA (II)*	25	3.20 (1.33)	29	3.33 (1.55)	0.707
*If I had no AAA, my health would be (17)*	25	−1.75 (3.25)	29	0.00 (3.25)	0.316

Abbreviations: AAA, abdominal aortic aneurysm; ADQoL, Aneurysm-Dependent Quality of Life questionnaire; n, number of patients; WI, weighted impact; SD, standard deviation; IQR, interquartile range; QoL, quality of life. For the individual evaluation lower mean values indicate superior quality of life. The data were analyzed with the Mann-Whitney U and Wilcoxon rank-sum tests. Bolded results denote statistically significant differences.

## Data Availability

The original contributions presented in this study are included in the article/Supplementary Material. Further inquiries can be directed to the corresponding author(s).

## Supplementary Materials

The following supporting information can be downloaded at: https://www.mdpi.com/article/10.3390/medsci13040273/s1; Table S1: Value transformation of the validated QoL questionnaires; Table S2: Item allocation to health concepts or subscales of QoL questionnaires; Table S3: Demographics of AAA patients with QoL data of the MetAAA trial with metformin or placebo intake; Table S4: Baseline characteristics of non-diabetic AAA patients with abdominal aortic aneurysm randomized to either the metformin group or placebo group (intention to treat cohort); Table S5: Absolute and relative frequencies of analyzed QoL questionnaires; Table S6: Detailed severity-graded answer frequencies of the ASRQ; Table S7: Individual evaluation of patient reported outcome measures of the ADQoL; Table S8: Comparison of the SF-36 health concepts, the ASRQ and ADQoL subscales between AAA patients of the MetAAA trial with metformin or placebo intake at baseline as a measure of health-related quality of life; Table S9: Comparison of the SF-36 health concepts, the ASRQ and ADQoL subscales between AAA patients of the MetAAA trial with metformin or placebo intake after twelve months of treatment as a measure of health-related quality of life; Table S10: Correlation of inflammatory blood parameters with quality of life patient reported outcomes.

## Abbreviations

The following abbreviations are used in this manuscript:

AAA	Abdominal aortic aneurysm
ADQoL	Aneurysm-Dependent Quality of Life
ASRQ	Aneurysm Symptom Rating Questionnaire
eGFR	Estimated glomerular filtration rate
IQR	Interquartile range
MAAAGI	Metformin for Abdominal Aortic Aneurysm Growth Inhibition
MetAAA	Metformin Therapy in Non-diabetic Patients with Abdominal Aortic Aneurysm
MPO	Myeloperoxidase
PROM	Patient-reported outcome measure
QoL	Quality of life
SD	Standard deviation
SF-36	36-Item Short Form Health Survey
