# Supplementary material for: MetAAA Trial Patients Receiving Metformin Therapy Show Limited Improvement in Quality of Life Compared to AAA Patients with Placebo Intake—A Double-Blind, Randomized, and Placebo-Controlled Trial
Source: Med Sci (Basel). 2025 Nov 15;13(4):273. doi: 10.3390/medsci13040273 (PMC12641964; doi:10.3390/medsci13040273)
Supplement: Supplementary file 1 [file medsci-13-00273-s001.zip › medsci-3942860-supplementary.pdf]

Supplementary Materials: MetAAA Trial Patients Receiving Metformin Therapy Show Limited Improvement in Quality of Life Compared to AAA Patients with Placebo Intake—A Double-Blind, Randomized, and Placebo-Controlled Trial

Supplemental Table S1: Value transformation of the validated QoL questionnaires.

Validated questionnaire	*Transformation of values*		
	Item number	*Original response category*	*Transformed value*
SF-36	1,2,20,22,34,36	1	100
		2	75
		3	50
		4	25
		5	0
	3,4,5,6,7,8,9,10,11,12	1	0
		2	50
		3	100
	13,14,15,16,17,18,19	1	0
		2	100
	21,23,26,27,30	1	100
		2	80
		3	60
		4	40
		5	20
		6	0
	24,25,28,29,31	1	0
		2	20
		3	40
		4	60
		5	80
		6	100
	32,33,35	1	0
		2	25
		3	50
		4	75
		5	100
ASRQ	1-44A	1	100
		2	0
	1-44B	1	0
		2	33
		3	66
		4	99
ADQoL	I,II	-	-
	2,4,6,8,9	1	0
		2	100
	PROM 1-22 Item A	1	-3
		2	-2
		3	-1
		4	0
		5	1
	PROM 1-22 Item B	1	3
		2	2
		3	1
		4	0

Abbreviations: SF-36, 36-Item Short Form Health Survey; ASRQ, Aneurysm Symptom Rating Questionnaire; ADQoL, Aneurysm-Dependent Quality of Life questionnaire; PROM, patient reported outcome measure.

Supplemental Table S2: Item allocation to health concepts or subscales of QoL questionnaires.

Validated questionnaire	*Health concepts or subscales*	*Number of items*	*Allocated items*
SF-36	General health perception	5	1,33,34,35,36
	Physical functioning	10	3,4,5,6,7,8,9,10,11,12
	Bodily pain	2	21,22
	Role limitations due to physical health problems	4	13,14,15,16
	Role limitations due to personal or emotional problems	3	17,18,19
	Emotional well-being	5	24,25,26,28,30
	Social functioning	2	20,32
	Energy/fatigue	4	23,27,29,31
	Health change over recent 12 months	1	2
ASRQ	General malaise	9	3,8,11,12,22,25,31,32,34
	Weight	3	35,36,37
	Emotional	9	13,14,15,16,17,28,29,30,33
	Lower limb	9	4,5,6,7,10,23,24,26,27
	Cognitive	6	1,2,18,19,20,21
	Gastrointestinal	8	9,38,39,40,41,42,43,44
ADQoL	Physical function	6	5,10,11,12,19,20
	Psychological health	3	14,21,22
	Social life	12	1,2,3,4,6,7,8,9,13,15,16,18
	Individual Evaluation	3	I,II,17

Abbreviations: SF-36, 36-Item Short Form Health Survey; ASRQ, Aneurysm Symptom Rating Questionnaire; ADQoL, Aneurysm-Dependent Quality of Life questionnaire.

Supplemental Table S3: Demographics of AAA patients with QoL data of the MetAAA trial with metformin or placebo intake.

Parameter			***MetAAA trial cohort Placebo (n=25)***		***MetAAA trial cohort Metformin (n=29)***
Continuous variables		*n/n ♂/♀*	*Median (IQR)*	n/n ♂/♀	*Median (IQR)*
Age [years]		22/3	71.00 (12.00)	22/7	72.00 (12.00)
Body mass index [kg/m²]		22/3	26.86 (2.59)	22/7	28.34 (6.38)
Smoking pack-years [py]		20/2	40.00 (22.38)	20/7	40.00 (36.60)
Mean systolic blood pressure [mmHg]		21/3	133.25 (18.00)	22/7	132.00 (10.00)
Mean diastolic blood pressure [mmHg]		21/3	79.50 (7.00)	22/7	78.50 (8.00)
Maximal AAA diameter [mm]		15/2	42.60 (5.60)	19/3	44.70 (8.90)
Aortic segment volume [cm³]		15/2	76.51 (56.71)	19/3	70.86 (54.82)
Maximal ILT diameter [mm]		15/2	14.00 (6.90)	19/3	9.80 (12.10)
ILT volume [cm³]		15/2	30.17 (29.99)	19/3	18.23 (43.08)
Percentage glycosylated hemoglobin (HbA_{1c}) [%]		22/3	5.65 (0.50)	22/7	5.50 (0.30)
Leukocytes [$x10^9/l$]		22/3	7.57 (2.21)	22/7	6.58 (2.97)
C-reactive protein [mg/dl]		22/3	0.13 (0.30)	22/7	0.24 (0.68)
D-dimer [µg/ml]		21/3	1.16 (0.85)	22/7	0.88 (1.04)
Myeloperoxidase [ng/ml]		22/3	19.77 (12.45)	21/7	18.63 (9.62)
D-dimer/Myeloperoxidase score		21/3	2.56 (2.73)	21/7	2.43 (2.79)
Nominal variables			*Frequency n (%)*		*Frequency n (%)*
Sex	*Men*		22 (88.00)		22 (75.86)
	Women		3 (12.00)		7 (24.14)
Smoking	*Never*	22/3	3 (12.00)	21/7	1 (3.45)
	Past	22/3	11 (44.00)	22/7	12 (41.38)
	Current	22/3	11 (44.00)	22/7	16 (55.17)
AAA family history		22/3	3 (12.00)	22/7	4 (13.79)
Hypertension		22/3	22 (88.00)	22/7	19 (65.52)
Hyperlipidemia		22/3	20 (80.0)	22/7	19 (65.52)
Coronary heart disease		22/3	9 (36.00)	22/7	5 (17.24)
Myocardial infarction		22/3	5 (20.00)	22/7	4 (13.79)
Stroke		22/3	2 (8.00)	22/7	1 (3.45)
Diabetes mellitus type II		22/3	0 (0.00)	22/7	0 (0.00)
Prediabetes		22/3	12 (48.00)	22/7	11 (37.90)
Peripheral artery disease		22/3	8 (32.00)	22/7	5 (17.24)
COPD		22/3	4 (16.00)	22/7	2 (6.90)

Antiplatelet therapy		22/3	21 (84.00)	22/7	24 (82.76)
Anticoagulation therapy		22/3	7 (28.00)	22/7	6 (20.69)
Antihypertensive therapy		22/3	24 (96.00)	22/7	22 (75.86)
Lipid-lowering agents		22/3	25 (100)	22/7	28 (96.55)
Antidiabetic medication		22/3	0 (0.00)	22/7	0 (0.00)
Metformin intake (not as IMP)		22/3	0 (0.00)	22/7	0 (0.00)
Educational level	*Primary school*	22/3	1 (4.00)	22/7	1 (3.45)
	Compulsory secondary or academic secondary school (lower cycle)	22/3	3 (12.00)	22/7	3 (10.34)
	Vocational school and apprenticeship	22/3	13 (52.00)	22/7	13 (44.83)
	Secondary technical and higher vocational school	22/3	3 (12.00)	22/7	5 (17.24)
	Academic secondary school (upper cycle)	22/3	1 (4.00)	22/7	3 (10.34)
	University or university of applied sciences	22/3	2 (8.00)	22/7	2 (6.90)

Abbreviations: n, number of individuals; AAA, abdominal aortic aneurysm; IQR, interquartile range; ILT, intraluminal thrombus; COPD, chronic obstructive pulmonary disease; IMP, investigational medical product.

Supplemental Table S4: Baseline characteristics of non-diabetic AAA patients with abdominal aortic aneurysm randomized to either the metformin group or placebo group (intention to treat cohort).

Parameter	***MetAAA trial cohort Placebo (n=28)***	***MetAAA trial cohort Metformin (n=30)***
Age [years]	69.86 ± 7.77	70.87 ± 9.7
Body mass index [kg/m²]	27.56 ± 3.01	28.96 ± 5.78
Male participants	25 (89%)	23 (77%)
Active smoking	12 (43%)	17 (57%)
History of smoking	12 (43%)	12 (40%)
Diabetes mellitus type II	1 (4%)	0 (0%)
Hypertension	24 (86%)	20 (67%)
Myocardial infarction	5 (18%)	5 (17%)
COPD	5 (18%)	3 (10%)
Lipid lowering agents	28 (100%)	28 (93%)
Anti platelet therapy	22 (79%)	25 (83%)
Maximal AAA diameter at baseline [mm]	88.44 ± 41.87	76.25 ± 40.55
Aortic segment volume at baseline [cm³]	43.57 ± 6.12	42.81 ± 6.38

Abbreviations: n, number of individuals; AAA, abdominal aortic aneurysm; COPD, chronic obstructive pulmonary disease; Data are presented as mean ± standard deviation or as n (%).

Supplemental Table S5: Absolute and relative frequencies of analyzed QoL questionnaires.

AAA study cohort	*Monitoring timepoint*	*QoL questionnaire*			
		Frequency n (%)			*Total*
		SF-36	*ASRQ*	*ADQoL*	
MetAAA trial Placebo	**Total**	**111 (33.94)**	**106 (32.42)**	**110 (33.64)**	**327 (49.62)**
	baseline	21 (35.59)	19 (32.20)	19 (32.20)	59 (18.04)
	3 months	21 (32.81)	21 (32.81)	22 (34.38)	64 (19.57)
	6 months	22 (34.38)	20 (31.25)	22 (34.38)	64 (19.57)
	9 months	23 (33.82)	22 (32.35)	23 (33.82)	68 (20.80)
	12 months	13 (33.33)	13 (33.33)	13 (33.33)	39 (11.93)
	18 months (follow-up)	2 (33.33)	2 (33.33)	2 (33.33)	6 (1.83)
		9 (33.33)	9 (33.33)	9 (33.33)	27 (8.26)
MetAAA trial Metformin	**Total**	**114 (34.34)**	**108 (32.53)**	**110 (33.13)**	**332 (50.38)**
	baseline	24 (34.29)	23 (32.86)	23 (32.86)	70 (21.08)
	3 months	23 (33.82)	22 (32.36)	23 (33.82)	68 (20.48)
	6 months	21 (36.21)	18 (31.03)	19 (32.76)	58 (17.47)
	9 months	20 (34.49)	19 (32.76)	19 (32.76)	58 (17.47)
	12 months	17 (33.33)	17 (33.33)	17 (33.33)	51 (15.36)
	18 months (follow-up)	3 (33.33)	3 (33.33)	3 (33.33)	9 (2.71)
		6 (33.33)	6 (33.33)	6 (33.33)	18 (5.42)
Combined cohorts	**Total**	**225 (34.14)**	**214 (32.47)**	**220 (33.38)**	**659 (100.00)**

Abbreviations: AAA, abdominal aortic aneurysm; QoL, quality of life; n, number of questionnaires; SF-36, 36-Item Short Form Health Survey; ASRQ, Aneurysm Symptom Rating Questionnaire; ADQoL, Aneurysm-Dependent Quality of Life questionnaire.

Supplemental Table S6: Detailed severity-graded answer frequencies of the ASRQ.

MetAAA trial<br>*Placebo*											
ASRQ subscales	**PROM**	**Symptom**	**Mean [%] (SD)**	**Total n**	**Symptoms n**	**Severity**					**Symptom occurrence [%]**
						**1**	**2**	**3**	**4**	**5**	
General malaise	3	Feverish	44.00 (19.05)	106	3	0	2	1	0	0	2.83
	8	Pain/discomfort back	65.27 (27.69)	106	45	1	13	17	14	0	42.45
	11	Excessive bruising	22.00 (35.41)	105	12	8	1	2	1	0	11.43
	12	Minor illness	59.40 (30.32)	106	10	0	5	2	3	0	9.43
	22	Dizzy/lightheaded	49.50 (22.17)	105	42	1	22	16	3	0	40.00
	25	Trembling	45.38 (26.61)	103	17	1	10	3	2	1	16.50
	31	Excessive sweating	56.57 (24.95)	105	8	0	3	3	1	1	7.62
	32	Episodes too hot/cold	40.76 (21.92)	106	18	2	9	6	0	1	16.98
	34	Generally weak	55.76 (23.51)	105	30	0	13	12	4	1	28.57
Weight	35	Poor appetite	37.13 (11.67)	106	9	0	7	1	0	1	8.49
	36	Lost weight	22.00 (26.94)	105	16	8	4	3	0	1	15.24
	37	Gained weight	44.00 (26.26)	105	21	2	12	5	2	0	20.00
Emotional	13	Depressed/low	48.71 (22.43)	106	21	1	10	9	1	0	19.81
	14	Feelings of panic	33.00 (33.00)	105	3	1	1	1	0	0	2.86
	15	Worried/nervous	47.67 (23.03)	106	27	1	15	9	2	0	25.47
	16	Irritable/angry	51.56 (24.01)	105	16	0	9	5	2	0	15.24
	17	Emotional/upset	39.35 (20.91)	105	26	2	18	5	1	0	24.76
	28	Lost interest in sex	55.00 (35.13)	100	23	3	7	5	6	2	23.00
	29	Avoid sexual activity	58.67 (32.07)	100	10	1	2	4	2	1	10.00
	30	Problems sexual function	68.64 (28.45)	100	28	1	5	10	9	3	28.00
	33	Sleep problems	59.58 (23.43)	106	38	0	13	17	6	2	35.85
Lower limb	4	Pain/discomfort in calves	70.46 (24.80)	105	37	0	8	16	13	0	35.24
	5	Pain/discomfort thighs	55.44 (26.47)	106	25	1	10	10	4	0	23.58
	6	Pain/discomfort groin	60.18 (24.01)	105	18	0	6	8	3	1	17.14
	7	Pain/discomfort buttocks	63.25 (22.06)	105	12	0	3	7	2	0	11.43
	10	Wound infection	55.00 (38.11)	105	3	0	2	0	1	0	2.86
	23	Tingling legs/feet	56.29 (27.45)	106	52	1	24	15	11	1	49.06
	24	Heaviness in legs	61.74 (25.19)	104	32	1	8	16	6	1	30.77
	26	Weakness in legs	68.44 (28.84)	105	28	1	6	10	10	1	26.67
	27	Swollen legs	54.58 (33.58)	106	26	4	7	9	6	0	24.53

Cognitive	1	Tired	67.14 (24.13)	106	30	0	7	14	8	1	28.30
	2	Headaches	45.38 (23.72)	105	16	1	9	5	1	0	15.24
	18	Difficulty concentrating	52.16 (26.64)	106	32	2	13	12	4	1	30.19
	19	Memory problems	55.00 (24.95)	106	37	1	15	15	5	1	34.91
	20	Difficulty thinking	48.84 (25.42)	106	26	1	14	7	3	1	24.53
	21	Unsteady/uncoordinated	38.82 (17.44)	105	17	1	12	4	0	0	16.19
Gastrointestinal	9	Abdominal pain	52.41 (26.24)	104	17	1	7	7	2	0	16.35
	38	Indigestion/heartburn	64.17 (17.80)	106	19	0	3	13	2	1	17.92
	39	Nausea/vomited	66.00 (46.67)	105	3	0	1	0	1	1	2.86
	40	Flatulence/belching	53.78 (29.16)	105	27	2	11	9	5	0	25.71
	41	Bloated	60.92 (26.42)	105	13	0	5	5	3	0	12.38
	42	Diarrhea	52.50 (28.18)	105	22	2	8	9	3	0	20.95
	43	Constipation	68.20 (23.22)	106	17	0	3	8	4	2	16.04
	44	Difficulty urinating	53.63 (35.00)	106	9	1	3	2	2	1	8.49

MetAAA trial
Metformin

ASRQ subscales	**PROM**	**Symptom**	**Mean [%] (SD)**	**Total n**	**Symptoms n**	**Severity**					**Symptom occurrence [%]**
						**1**	**2**	**3**	**4**	**5**	
General malaise	3	Feverish	57.75 (31.60)	108	4	0	2	1	1	0	3.70
	8	Pain/discomfort back	63.46 (25.18)	108	53	2	11	28	11	1	49.07
	11	Excessive bruising	38.08 (22.73)	108	13	2	7	4	0	0	12.04
	12	Minor illness	52.80 (23.07)	108	11	1	2	7	0	0	10.19
	22	Dizzy/lightheaded	55.00 (24.22)	108	27	0	13	10	4	0	25.00
	25	Trembling	46.20 (29.52)	108	5	0	4	0	1	0	4.63
	31	Excessive sweating	54.00 (33.07)	108	22	3	7	7	5	0	20.37
	32	Episodes too hot/cold	68.36 (27.35)	108	14	0	4	5	5	0	12.96
	34	Generally weak	56.10 (28.94)	108	30	1	14	8	7	0	27.78
Weight	35	Poor appetite	25.38 (19.77)	108	13	4	8	1	0	0	12.04
	36	Lost weight	7.86 (17.79)	108	21	17	3	1	0	0	19.44
	37	Gained weight	57.75 (35.13)	108	16	2	5	4	5	0	14.81
Emotional	13	Depressed/low	58.38 (23.44)	106	27	0	10	12	4	0	25.47
	14	Feelings of panic	66.00 (26.94)	107	7	0	2	3	2	0	6.54
	15	Worried/nervous	57.12 (22.00)	108	26	0	10	13	3	0	24.07
	16	Irritable/angry	55.85 (24.79)	108	13	0	6	5	2	0	12.04
	17	Emotional/upset	54.35 (25.93)	108	17	0	9	5	3	0	15.74

	28	Lost interest in sex	37.89 (27.00)	103	28	7	9	11	0	1	27.18
	29	Avoid sexual activity	57.00 (15.41)	101	12	0	3	8	0	1	11.88
	30	Problems sexual function	50.16 (21.56)	97	28	1	11	12	1	3	28.87
	33	Sleep problems	54.12 (18.76)	108	25	0	10	14	1	0	23.15
Lower limb	4	Pain/discomfort in calves	70.89 (27.00)	108	28	0	7	9	11	1	25.93
	5	Pain/discomfort thights	83.77 (21.79)	108	13	0	1	4	8	0	12.04
	6	Pain/discomfort groin	67.74 (23.27)	108	19	0	4	10	5	0	17.59
	7	Pain/discomfort buttocks	71.08 (22.73)	108	13	0	2	7	4	0	12.04
	10	Wound infection	33.00 (00.00)	108	1	0	1	0	0	0	0.93
	23	Tingling legs/feet	56.29 (26.36)	108	35	1	14	13	6	1	32.41
	24	Heaviness in legs	66.00 (28.00)	108	26	1	6	11	8	0	24.07
	26	Weakness in legs	72.29 (30.64)	108	21	1	4	6	10	0	19.44
	27	Swollen legs	62.59 (32.22)	108	29	2	9	8	10	0	26.85
Cognitive	1	Tired	66.00 (28.94)	108	27	1	7	10	9	0	25.00
	2	Headaches	42.43 (16.10)	106	7	0	5	2	0	0	6.60
	18	Difficulty concentrating	52.41 (31.00)	108	17	1	9	3	4	0	15.74
	19	Memory problems	45.38 (26.61)	108	16	2	7	6	1	0	14.81
	20	Difficulty thinking	46.20 (27.83)	108	10	1	5	3	1	0	9.26
	21	Unsteady/uncoordinated	55.00 (23.33)	108	9	0	4	4	1	0	8.33
Gastrointestinal	9	Abdominal pain	67.83 (28.80)	107	18	0	6	5	7	0	16.82
	38	Indigestion/heartburn	55.00 (29.29)	108	12	1	4	5	2	0	11.11
	39	Nausea/vomited	99.99 (00.00)	108	1	0	0	0	1	0	0.93
	40	Flatulence/belching	62.33 (30.81)	108	28	2	7	10	8	1	25.93
	41	Bloated	68.44 (28.84)	108	27	1	6	10	10	0	25.00
	42	Diarrhea	54.00 (23.98)	108	22	0	11	8	3	0	20.37
	43	Constipation	62.70 (28.89)	107	10	0	4	3	3	0	9.35
	44	Difficulty urinating	49.50 (70.00)	107	2	1	0	0	1	0	1.87

Abbreviations: AAA, abdominal aortic aneurysm; PROM, patient reported outcome measure; SD, standard deviation; total n, number of questionnaires; symptoms n, number of reported symptoms; Severity: 1 = not relevant, 2 = low, 3 = moderate, 4 = high, 5 = unknown.

Supplemental Table S7: Individual evaluation of patient reported outcome measures of the ADQoL.

		MetAAA trial cohort Placebo		*MetAAA trial cohort Metformin*		*p value*
ADQoL subscale	**Domain**	*n*	*Score (SD)*	*n*	*Score (SD)*	
Physical function	Physical ability	105	-1.58 (2.21)	108	-1.55 (2.45)	0.355
	Getting out and about	105	-0.17 (2.49)	107	-0.50 (3.02)	0.786
	Household tasks	104	-0.33 (2.54)	109	-0.32 (3.04)	0.438
	Being able to do things for others	102	-0.24 (2.71)	109	-0.08 (2.60)	0.670
	Energy	72	-1.64 (2.43)	72	-1.82 (2.77)	0.941
	Physical discomfort	72	-1.86 (2.66)	72	-1.75 (2.48)	0.793
Psychological health	Feelings about the future	71	-2.24 (2.62)	73	-1.95 (2.76)	0.240
	Anxiety	72	-2.63 (2.97)	73	-2.15 (2.87)	0.263
	Ability to think clearly, concentrate and/or remember things	72	-1.43 (2.53)	73	-1.01 (2.03)	0.374
Social life	Leisure	108	-1.21 (1.77)	109	-1.67 (2.35)	0.397
	Working life	38	-0.68 (1.34)	35	-1.26 (2.36)	0.371
	Local or long-distance journeys	104	-1.08 (2.07)	109	-1.40 (2.27)	0.545
	Holidays	83	-1.23 (2.22)	89	-1.52 (2.38)	0.434
	Family life	101	-0.25 (2.60)	105	-0.44 (2.96)	0.931
	Friendships and social life	99	-0.19 (2.34)	103	-0.50 (2.70)	0.728
	Personal relationships	90	0.24 (2.38)	95	-0.27 (3.22)	0.674
	Sex life	82	-0.10 (2.52)	91	-0.01 (2.72)	0.583
	Enjoyment of food	72	-0.60 (1.60)	73	-0.93 (2.00)	0.288
	Financial situation	73	-0.79 (2.32)	73	-0.63 (1.67)	0.746
	Dependence of others	71	-1.15 (2.42)	71	-1.25 (2.61)	0.949
	The amount people fuss or worry about me	71	-2.25 (3.16)	71	-1.93 (3.07)	0.387
Individual evaluation	If I had no AAA, my health would be	72	-2.49 (3.13)	73	-2.56 (2.79)	0.616
Total	Average weighted impact	108	-0.73 (1.97)	110	-0.77 (2.19)	0.523

Abbreviations: AAA, abdominal aortic aneurysm; ADQoL, Aneurysm-Dependent Quality of Life questionnaire; n, number of questionnaires; SD, standard deviation. The following statistical analysis was applied: Mann-Whitney U test.

Supplemental Table S8: Comparison of the SF-36 health concepts, the ASRQ and ADQoL subscales between AAA patients of the MetAAA trial with metformin or placebo intake at baseline as a measure of health-related quality of life.

	MetAAA trial cohort Placebo		***MetAAA trial cohort Metformin***		***p value***
SF-36 – Health concepts (at baseline)	*n*	*Mean [%] (SD)*	*n*	*Mean [%] (SD)*	
General health perception	21	57.08 (17.10)	24	58.13 (19.61)	0.673
Physical functioning	21	67.33 (22.52)	24	60.42 (29.34)	0.561
Bodily pain	20	74.38 (23.05)	24	67.08 (30.00)	0.455
Role limitations due to physical health problems	19	46.05 (45.08)	24	59.38 (42.87)	0.345
Role limitations due to personal or emotional problems	19	66.67 (41.57)	23	69.57 (43.71)	0.636
Emotional well-being	20	75.60 (16.72)	23	74.78 (17.54)	0.855
Social functioning	20	85.00 (18.41)	24	81.25 (20.52)	0.553
Energy/fatigue	20	53.75 (17.91)	23	59.35 (19.67)	0.207
Health change over recent 12 months	21	53.57 (22.76)	24	53.13	0.949
ASRQ subscales (at baseline)	***MetAAA trial cohort Placebo***		***MetAAA trial cohort Metformin***		***p value***
Appearance of negative symptoms	*n/N of items*	*%*	*n/N of items*	*%*	
General malaise	42/168	25.00	46/207	22.22	0.528
Weight	5/55	9.09	6/69	8.70	0.939
Emotional	36/167	21.56	49/204	24.02	0.410
Lower limb	45/165	27.27	49/207	23.67	0.427
Cognitive	27/114	23.68	25/137	18.25	0.290
Gastrointestinal	27/151	17.88	27/182	14.84	0.453
Total appearance of negative symptoms	182/783	23.24	200/961	20.81	0.202
Other negative symptoms (non-assignable)	0/21	0.00	2/24	8.33	-
Limitations due to symptoms	*n (of questionnaires)*	*Mean [%] (SD)*	*n (of questionnaires)*	*Mean [%] (SD)*	
General malaise	19	2.37 (1.61)	23	2.00 (1.71)	0.426
Weight	19	0.37 (0.76)	23	0.26 (0.45)	0.908
Emotional	19	2.47 (2.29)	23	2.43 (1.93)	0.878
Lower limb	19	2.68 (2.21)	23	2.13 (2.32)	0.396
Cognitive	19	1.42 (1.43)	23	1.13 (1.49)	0.451
Gastrointestinal	19	1.47 (1.78)	23	1.26 (1.29)	0.927
Total limitations due to symptoms	19	11.79 (6.25)	23	10.22 (5.95)	0.288
ADQoL subscales (at baseline)	***MetAAA trial cohort Placebo***		***MetAAA trial cohort Metformin***		***p value***

	n	WI (SD)	n	WI (SD)	
Physical function	18	-1.75 (2.40)	23	-1.86 (2.45)	0.806
Psychological health	17	-2.21 (2.27)	23	-1.83 (2.36)	0.340
Social life	19	-1.10 (1.89)	23	-1.59 (2.32)	0.718
Individual evaluation	***MetAAA trial cohort Placebo***		***MetAAA trial cohort Metformin***		***p value***
	n	Mean (SD)	n	Mean (SD)	
Current QoL (I)	19	4.26 (0.65)	23	4.43 (0.66)	0.363
QoL if I would not have an AAA (II)	19	3.05 (0.97)	22	3.00 (1.11)	0.978
If I had no AAA, my health would be (17)	17	-3.47 (3.87)	23	-2.26 (2.72)	0.441

Abbreviations: Abbreviations: AAA, abdominal aortic aneurysm; SF-36, 36-Item Short Form Health Survey; n, number of questionnaires; SD, standard deviation; ASRQ, Aneurysm Symptom Rating Questionnaire; n, number of applicable items; N, total number of items; Scores represent the percentage of total possible score achieved. ADQoL, Aneurysm-Dependent Quality of Life questionnaire; n, number of questionnaires; WI, weighted impact; QoL, quality of life. For the individual evaluation lower mean values indicate superior quality of life. The following statistical analyses were applied: χ^2 test and Mann-Whitney U test.

Supplemental Table S9: Comparison of the SF-36 health concepts, the ASRQ and ADQoL subscales between AAA patients of the MetAAA trial with metformin or placebo intake after twelve months of treatment as a measure of health-related quality of life.

	MetAAA trial cohort Placebo		***MetAAA trial cohort Metformin***		***p value***
SF-36 – Health concepts (after 12 months of treatment)	*n*	*Mean [%] (SD)*	*n*	*Mean [%] (SD)*	
General health perception	13	55.10 (13.23)	17	59.41 (24.30)	0.483
Physical functioning	13	63.46 (18.41)	17	67.94 (34.69)	0.170
Bodily pain	13	80.38 (25.61)	17	75.88 (29.91)	0.837
Role limitations due to physical health problems	13	55.77 (41.02)	17	63.24 (45.17)	0.432
Role limitations due to personal or emotional problems	13	82.05 (32.25)	17	78.43 (37.16)	0.967
Emotional well-being	13	79.15 (18.64)	17	74.82 (29.08)	1.000
Social functioning	13	73.08 (26.93)	17	83.82 (23.70)	0.229
Energy/fatigue	13	54.62 (20.76)	17	63.24 (25.80)	0.183
Health change over recent 12 months	13	46.15 (13.87)	16	51.56 (6.25)	0.374
ASRQ subscales (after 12 months of treatment)	***MetAAA trial cohort Placebo***		***MetAAA trial cohort Metformin***		***p value***
Appearance of negative symptoms	*n/N of items*	*%*	*n/N of items*	*%*	
General malaise	42/117	35.90	59/153	38.56	0.654
Weight	12/39	30.77	21/51	41.18	0.310
Emotional	39/115	33.91	61/145	42.07	0.179

Lower limb	51/117	43.59	61/153	39.87	0.539
Cognitive	33/78	42.31	34/101	33.66	0.236
Gastrointestinal	40/104	38.46	47/136	34.56	0.533
Total appearance of negative symptoms	210/544	38.60	270/704	38.35	0.928
Other negative symptoms (non-assignable)	5/13	38.46	4/17	23.53	0.198
Limitations due to symptoms	*n (of questionnaires)*	*Mean [%] (SD)*	*n (of questionnaires)*	*Mean [%] (SD)*	
General malaise	13	6.33 (7.44)	17	8.27 (11.04)	0.837
Weight	13	1.00 (3.03)	17	5.29 (9.56)	0.300
Emotional	13	3.08 (4.65)	17	5.67 (9.32)	0.934
Lower limb	13	9.62 (10.73)	17	10.65 (18.50)	0.483
Cognitive	13	8.50 (12.13)	17	3.74 (6.37)	0.229
Gastrointestinal	13	5.31 (8.81)	17	4.36 (8.89)	0.711
Total limitations due to symptoms	13	8.06 (6.19)	17	7.96 (8.39)	0.711
ADQoL subscales (after 12 months of treatment)	***MetAAA trial cohort Placebo***		***MetAAA trial cohort Metformin***		***p value***
	n	WI (SD)	n	WI (SD)	
Physical function	13	-1.41 (1.28)	17	-1.20 (1.94)	0.341
Psychological health	13	-2.00 (2.35)	17	-1.28 (1.85)	0.563
Social life	13	-0.95 (1.03)	17	-0.88 (1.32)	0.509
Individual evaluation	***MetAAA trial cohort Placebo***		***MetAAA trial cohort Metformin***		***p value***
	n	Mean (SD)	n	Mean (SD)	
Current QoL (I)	11	3.18 (1.17)	17	3.29 (1.31)	0.890
QoL if I would not have an AAA (II)	11	2.82 (0.75)	17	3.12 (1.11)	0.306
If I had no AAA, my health would be (17)	4	-3.75 (3.86)	5	-2.40 (1.52)	0.730

Abbreviations: Abbreviations: AAA, abdominal aortic aneurysm; SF-36, 36-Item Short Form Health Survey; n, number of questionnaires; SD, standard deviation; ASRQ, Aneurysm Symptom Rating Questionnaire; n, number of applicable items; N, total number of items; Scores represent the percentage of total possible score achieved. ADQoL, Aneurysm-Dependent Quality of Life questionnaire; n, number of questionnaires; WI, weighted impact; QoL, quality of life. For the individual evaluation lower mean values indicate superior quality of life. The following statistical analyses were applied: χ^2 test and Mann-Whitney U test.

Supplemental Table S10: Correlation of inflammatory blood parameters with quality of life patient reported outcomes.

	ADQoL subscales		
Parameter	Current QoL (I)	Physical function	Social life
	n (Spearman's rho)<br>p value		
Leukocytes [x10⁹/l]	54 (0.270)<br>**0.049**	-	-
C-reactive protein [mg/dl]	54 (0.314)<br>**0.021**	-	-
Myeloperoxidase [ng/ml]	-	53 (-0.409)<br>**0.002**	53 (-0.284)<br>**0.039**

Abbreviations: ADQoL, Aneurysm-Dependent Quality of Life questionnaire; n, number of measurements. For analyses of correlations, the Spearman's rank correlation coefficient (Spearmans's rho) was calculated. For the individual evaluation (Current QoL (I)) lower values indicate superior quality of life. Only significant correlations (boldface p values) are displayed in the table.
